# Acute Seronegative *Toxoplasma gondii* Hepatitis Allergic to First-Line Treatment

**DOI:** 10.1155/2018/1951936

**Published:** 2018-04-01

**Authors:** Fernando De la Garza-Salazar, Carlos Alejandro Cortéz-Hernández, Horacio Decanini-Arcaute, Adrián Camacho-Ortiz

**Affiliations:** ^1^Department of Internal Medicine, Christus Muguerza Hospital Alta Especialidad, Universidad de Monterrey, San Pedro Garza García, NL 64060, Mexico; ^2^Department of Gastroenterology, Hospital Universitario Dr. José Eleuterio González, Universidad Autónoma de Nuevo León, San Nicolás de los Garza, NL 64460, Mexico; ^3^Department of Pathology, Christus Muguerza Hospital Alta Especialidad, Universidad de Monterrey, San Pedro Garza García, NL 64060, Mexico; ^4^Epidemiologic Hospital Coordination, Hospital Universitario Dr. José Eleuterio González, Universidad Autónoma de Nuevo León, San Nicolás de los Garza, NL 64460, Mexico

## Abstract

*Toxoplasma gondii* infects up to one-third of the world's population, making it the protozoan that most infects people worldwide. Among the forms of presentation of toxoplasmosis, hepatitis is probably the least frequent with only a few case reports in the world's medical literature. Despite the fact that the first diagnostic test is the serology using the ELISA technique because its sensitivity is close to 100% in our case, it was reported negative. The biopsy gave the definitive diagnosis, and we were able to start treatment immediately. Although the first line of treatment is sulfadiazine and pyrimethamine, our sulfate allergic patient received an alternative regimen of doxycycline, clindamycin, and azithromycin with a good response. This is the only case of *Toxoplasma* hepatitis reported in Mexico and the only one in the world with negative serology with a good response to second-line treatment.

## 1. Introduction

Toxoplasmosis is a zoonosis caused by the protozoan *Toxoplasma gondii* which is considered to be the most frequent protozoan infection worldwide [1, 2]. Studies have shown that, in Mexico, about 32–62.6% of the population has been in contact with the parasite at least once and that the most frequent course of the disease is chronic and asymptomatic [3, 4]. The clinical scenario varies from person to person, where hepatitis is one of the least common forms of presentation [5–9]. The following is a case report where hepatitis was a diagnosis secondary to toxoplasmosis.

## 2. Case Presentation

An 18-year-old man arrives to the emergency room because of malaise, fever, pain in the right upper quadrant, and progressive jaundice. With exception of sulfas allergy, he reported no other medical history. He referred that during the last month he was exposed to cattle and swam in a river during a trip to a suburban area and consumed raw beef meat. In addition, the patient cohabited with 8 cats for 5 months and consumed energy drinks daily for 3 months.

The vital signs upon admission were normal. Neurological, cardiac, and pulmonary examinations were normal, and neither lymphadenopathy, hepatomegaly, nor splenomegaly was found; nevertheless, generalized jaundice and abdominal pain in the upper quadrant were found, whereas the rest of the physical exam had no abnormal findings.

Laboratory studies reported the following: hemoglobin 15.4 g/dL, leucocytes 7850/mm^3^ (differential count: 4980 neutrophils/mm^3^, 1820 lymphocytes/mm^3^, 940 monocytes/mm^3^, and 50 eosinophils/mm^3^ without atypical cells), platelets 239000/mm^3^, glucose 81 mg/dL, total cholesterol 109 mg/dL, triglycerides 179 mg/dL, total bilirubin 12.07 mg/dL with 10.37 mg/dL of direct bilirubin and 1.70 mg/dL of indirect bilirubin, aspartate transaminase (AST) 2050 IU/L (normal: 0–40 IU/L), alanine transaminase (ALT) 2777 IU/L (normal: 0–41 IU/L), alkaline phosphatase 198 IU/L (normal: 40–130 IU/L) with gamma-glutamyltransferase (GGT) 105 IU/L (normal: 8–61 IU/L), and lactic dehydrogenase 960 IU/L (normal: 8–61 IU/L). Total proteins, albumin, globulin, renal function, and serum electrolytes were within normal ranges. The main laboratory findings are shown in Table 1.

Abdominal ultrasound reported neither hepatomegaly, steatosis, nor dilatation of biliary tract; this was complemented with a magnetic cholangioresonance that did not find alterations.

Serological tests were performed, where the following results were negative: IgM antibody for hepatitis A, superficial hepatitis B antigen, anti-IgM antibodies against hepatitis B, anti-hepatitis C antibodies, and IgG and IgM antibodies against hepatitis E; others such as IgG and IgM antibodies against herpes virus 1 were reported in 2.19 and 0.07 index (negative: lower 0.6 and 0.8 index, resp.), IgG and IgM antibodies for herpes virus 2 in 0.406 and 0.85 index (negative: lower 0.51 and 0.8, resp.), heterophile antibodies (Paul–Bunnell) negative, IgM against Epstein–Barr virus capsid negative, Epstein–Barr virus DNA by quantitative PCR undetected (negative: <200 copies/mL), cytomegalovirus by quantitative PCR undetected (negative: <2.3 copies/mL), IgG and IgM antibodies against cytomegalovirus at 90.6 AU/mL and 0.11 AU/mL (negative: <6.0 AU/mL and <0.85 AU/mL, resp.), ELISA against HIV negative, IgG antibodies titles against *Fasciola hepatica* were 1 : 2 (negative < 1 : 32), anti-*Toxocara* antibodies negative, *Rickettsia rickettsii* and *Rickettsia typhi* IgG and IgM antibodies undetected, undetected *Leptospira* in dark-field microscopy in urine, and IgG and IgM in phase I and phase II for *Coxiella burnetii* negative. The determination of liver kidney microsomal antibodies was less than 20 IU (negative <20 IU), antibodies to soluble liver's antigen were negative (<20 IU), antinuclear antibodies by fluorescence were negative, and smooth muscle antibodies were <1 : 20 (negative). IgG and IgM antibodies against *Toxoplasma* reported the following results: 0.2 IU/mL (negative: <1.6 IU/mL) and 0.18 IU/mL (negative < 0.5 IU/mL), respectively. Three days later, to rule out a false negative, new antibody titers against *Toxoplasma* were ordered which reported 0.2 IU/mL for both IgG and IgM antibodies.

**Figure 1:**
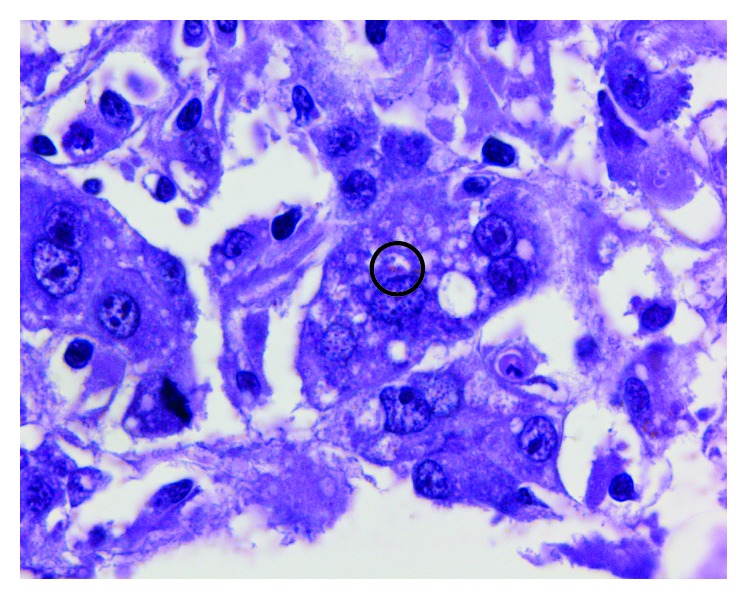
Histological findings. Black circle: liver biopsy stained with haematoxylin and eosin showing *Toxoplasma gondii* tachizoites (×40).

**Figure 2:**
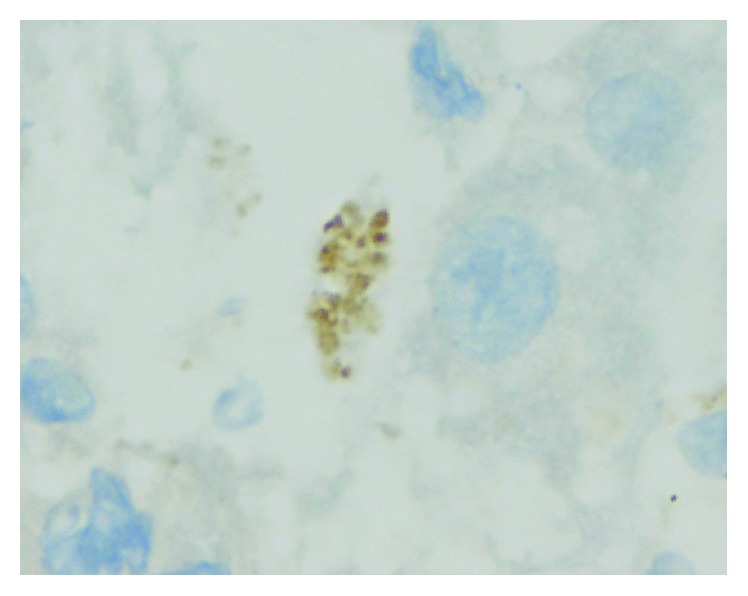
Immunohistochemistry findings. *Toxoplasma gondii* tachizoites in hepatocyte (×100).

In addition, the patient underwent a liver biopsy guided by ultrasound without complications. The biopsy findings and immunohistochemical panel are reported in Figures 1 and 2. We used a *Toxoplasma gondii* rabbit polyclonal antibody manufactured by Cell Marque (ref. 220A-18-ASR) and a polymer that reacts with diaminobenzidine (DAB); a positive control with brain tissue infected with *Toxoplasma* was used.

Once the biopsy was reported, in correlation with the clinical evolution and laboratory results, the patient was diagnosed with acute hepatitis secondary to *Toxoplasma gondii*; therefore, treatment was immediately started (two days before leaving the hospital) with doxycycline 100 mg PO BID, clindamycin 600 mg PO TID, and azithromycin 500 mg PO QD for 10 days. After its emplacement, the patient showed a favorable clinical response.

## 3. Discussion

*Toxoplasma gondii* is a protozoan transmitted through the ingestion of poorly cooked meat with cysts and contaminated food or water with oocysts eliminated through cat feces. In our patients' case, the two forms of transmission were probable [10]. The incubation period may vary; most of the reported cases refer the exposure one or two months prior to the onset of symptoms as in this given case. The clinical presentation of toxoplasmosis may vary; in immunocompetent individuals, the infection is asymptomatic in up to 80% of the cases and bilateral cervical lymphadenopathy is the most common presentation [10]. Some other less frequent forms of presentation are described such as encephalitis, retinitis, pneumonitis, myocarditis, myositis, and in rare cases acute hepatitis. These seldom forms of presentation usually occur in immunocompromised patients (HIV-positive and posttransplant recipients) and only as anecdotal cases in immunocompetent patients [5–9]. Most of the cases of hepatitis secondary to *Toxoplasma gondii* have been reported in Brazil, El Salvador, Turkey, and France, among others. Furthermore, as a common finding in all cases, eosinophilia and/or lymphoid atypia were reported, whereas in most cases cervical lymphadenopathy was found, and in few cases, hepatitis was the only clinical manifestation [10–13]. In our patient, these findings were never reported in the laboratories, despite the abundant eosinophilia and lymphoid infiltration in the liver biopsy.

There are several serological methods for the diagnosis of toxoplasmosis including indirect hemagglutination, indirect immunofluorescence, ELISA, and more recently western blotting as well as recently developed PCR techniques [14]. The ELISA method for IgM, IgG, and IgA antibodies against *Toxoplasma* is a good initial test since it has a sensitivity and specificity of 100% and 98.4%, respectively [2, 15]. In the cases of acute *Toxoplasma*-related hepatitis reported by Atilla et al. [10] and Doğan et al. [9], the diagnosis was made using high IgM titers which supported the recent exposure to the parasite. IgM is the first immunoglobulin to appear in serum, usually one week after infection with a peak at 1–3 months and a progressive decline until reported negative at 9 months. IgA has similar kinetics than IgM although the peak is slightly later and persists elevated only for 3-4 months. IgG appears in serum at the second week of infection with a peak at 3 months and remains in plateau from 6 months to throughout the individual's life (probably due to the persistence of latent cysts). In our patient, the determination of IgM and IgG immunoglobulin levels by ELISA was normal despite having clinical manifestations of more than 7 days of evolution, and as far as we know, there is no case reported in the current medical literature regarding acute hepatitis secondary to *Toxoplasma* with negative serology. A diagnostic algorithm based on serology adapted by Villard et al. in 2011 mentions that normal *Toxoplasma* levels of IgG and IgM rule out the possibility of a recent infection (7 days or more) [16]. The most frequent cause of false negatives in the determination of IgM against *Toxoplasma* is the neutralization of the antibodies by IgG immunoglobulin [9]. When a false negative is suspected, some authors suggest measuring IgA levels against *Toxoplasma*, although this approach is currently controversial [9, 17]. Given the failure to find by serological studies, the cause of the rapid and progressive liver function deterioration (repeated hypoglycemia, bilirubin elevation, elevation of transaminases, and prolongation of clotting times), a liver biopsy was performed in order to reach a definitive diagnosis and be able to start treatment in a timely manner. In the case of acute hepatitis secondary to *Toxoplasma*, there is not that much information regarding the treatment regimens, and some authors recommend that antibiotics should not be used at first instance because in most patients the condition is self-limiting [9, 12, 13]. Other authors recommend the administration of antibiotics only when there are complications or persistence of symptomatology for weeks, although exact temporality is not specified. In our patient, the persistence of symptoms, biochemical alterations, and the development of complications of acute liver failure gave the guideline to initiate the administration of antibiotics. The use of pyrimethamine with sulfadiazine plus folinic acid has been one of the most used treatments in toxoplasmosis and is considered as first-line treatment [18]. The toxicity of this combination (mainly allergic reactions, drug hepatitis, and hematological toxicity) limits their use. Another antibiotic scheme that can be used in case of lack of availability or tolerance to pyrimethamine is trimethoprim with sulfamethoxazole, which has a similar efficacy and same rate of side effects as first-line treatment [18]. The two previously mentioned schemes could not be used in our patient due to his sulfa allergy and the lack of availability of pyrimethamine in Mexico, so it was necessary to use an alternate scheme (clindamycin, doxycycline, and azithromycin). He was discharged completely asymptomatic and with improvement in the biochemical parameters.

## Figures and Tables

**Table 1:** Patient laboratory results during and after treatment.

	At admission	The day before biopsy	First day with antibiotics	The day of discharge
Glucose (mg/dL)	81	86	90	78
Cr (mg/dL)	0.9	0.7	0.7	0.7
Cholesterol (mg/dL)	109	91	103	93
Triglycerides (mg/dL)	179	194	160	159
Total bilirubin (mg/dL)	12.07	23.55	22.09	17.02
Direct bilirubin (mg/dL)	10.37	21.13	19.34	14.3
Albumin (g/dL)	4.7	3.8	3.7	3.2
AST/ALT (IU/L)	2050/2777	2799/3156	2219/2519	749/1053
ALP (IU/L)	198	147	137	137
GGT (IU/L)	105	36	37	30
LDH (IU/L)	960	1042	834	497

Cr: creatinine; ALP: alkaline phosphatase; GGT: gamma-glutamyltransferase; AST: aspartate aminotransferase; ALT: alanine aminotransferase; LDH: lactate dehydrogenase.
